# Recent Advances in Pharmacological and Non-Pharmacological Strategies of Cardioprotection

**DOI:** 10.3390/ijms20164002

**Published:** 2019-08-16

**Authors:** Afonso Caricati-Neto, Paolo Ruggero Errante, Francisco Sandro Menezes-Rodrigues

**Affiliations:** Department of Pharmacology, Laboratory of Autonomic and Cardiovascular Pharmacology, Escola Paulista de Medicina–Universidade Federal de São Paulo (UNIFESP), Vila Clementino, São Paulo-SP 04039-032, Brazil

**Keywords:** cardioprotection, ischemic conditioning, cardioprotective drugs, cardiac ischemia and reperfusion

## Abstract

Ischemic heart diseases (IHD) are the leading cause of death worldwide. Although the principal form of treatment of IHD is myocardial reperfusion, the recovery of coronary blood flow after ischemia can cause severe and fatal cardiac dysfunctions, mainly due to the abrupt entry of oxygen and ionic deregulation in cardiac cells. The ability of these cells to protect themselves against injury including ischemia and reperfusion (I/R), has been termed “cardioprotection”. This protective response can be stimulated by pharmacological agents (adenosine, catecholamines and others) and non-pharmacological procedures (conditioning, hypoxia and others). Several intracellular signaling pathways mediated by chemical messengers (enzymes, protein kinases, transcription factors and others) and cytoplasmic organelles (mitochondria, sarcoplasmic reticulum, nucleus and sarcolemma) are involved in cardioprotective responses. Therefore, advancement in understanding the cellular and molecular mechanisms involved in the cardioprotective response can lead to the development of new pharmacological and non-pharmacological strategies for cardioprotection, thus contributing to increasing the efficacy of IHD treatment. In this work, we analyze the recent advances in pharmacological and non-pharmacological strategies of cardioprotection.

## 1. Introduction

Ischemic heart diseases (IHD) are the main cause of death in developed and non-developed countries [1]. Although the principal form of treatment of IHD is myocardial reperfusion, the recovery of coronary blood flow after ischemia can cause cardiac dysfunctions, mainly due to the abrupt entry of oxygen and ionic deregulation in cardiac cells [2]. These dysfunctions produced by ischemia and reperfusion (I/R) injury may result in the collapse of myocardial function, increasing the incidence of cardiac arrhythmias and causing death of cardiac cells, due mainly to cytosolic Ca^2+^ overload, a deficit in ATP production by mitochondria and the formation of excessive free radicals [3]. As this cardiac collapse can be fatal, several non-pharmacological and pharmacological cardioprotective strategies have been proposed to protect the myocardium of lesions caused by I/R injury and increase the effectiveness of IHD treatment. In this work, we analyze the recent advances in non-pharmacological and pharmacological strategies for cardioprotection.

### 1.1. Cellular Signalling Involved in the Regulation of Cardiac Function

The mechanical activity of the heart is induced by electrical stimulus generated by the sinoatrial node (SA) and propagated by the atrioventricular node (AV) and His-Purkinje fibers to ventricles [4]. The cardiac electrical activity defines the format and the time of propagation of electrical signals detected by electrocardiogram (ECG) [4]. The electrical activity resulting from the propagation of action potential (AP) on the plasma membrane of cardiac cells reaches the T tubules stimulating the Ca^2+^ influx through L-type voltage-dependent Ca^2+^ channels (VDCC) [5]. The Ca^2+^ influx stimulates the Ca^2+^ release from sarcoplasmic reticulum (SR) through the activation of Ca^2+^ channels regulated by the ryanodine receptors (RyR) [5]. This Ca^2+^-release Ca^2+^-induced (CICR) mechanism transiently amplifies the cytosolic Ca^2+^ concentration ([Ca^2+^]c), which stimulates the activity of contractile proteins, unleashing the contraction of cardiac cells [5,6]. The free Ca^2+^ in the cytosol binds to troponin C (TnC), which favors the interaction of these proteins with troponin I (TnI), causing TnI to shift from the active site of actin, allowing the displacement of tropomyosin T (TmT) and troponin T (TnT), the myosin-actin interaction and the contraction of cardiac cells (systole) [6,7]. The increase of [Ca^2+^]c during the contraction of cardiac cells is restored to basal levels (resting) by Ca^2+^ sequestration SR and other cytoplasmic organelles, and Ca^2+^ extrusion by ionic transporters located in plasma membrane of cardiac cells [5]. This reduction in [Ca^2+^]c promotes the relaxation of cardiac cells (diastole) [5,8].

The SR has an important role in the regulating contraction-relaxation cycle in cardiac cells, acting as cytoplasmic reservoir of Ca^2+^ [9]. SR sequestrates the Ca^2+^ from cytosol by ATP-dependent Ca^2+^-ATPase (SERCA), especially by SERCA2_A_ [9]. The excess of Ca^2+^ in the cytosol is regulated by two Ca^2+^ extrusion mechanisms located in plasma membrane: the Na^+^/Ca^2+^ exchanger (NCX) and plasma membrane Ca^2+^-ATPase (PMCA) [5,9]. The NCX transports Ca^2+^ from cytosol to extracellular medium (reverse mode) or from extracellular medium to cytosol (reverse mode) [9,10]. Thus, SERCA, NCX and PCMA play an important role in the contraction-relaxation cycle by finely regulating the [Ca^2+^]c in cardiac cells.

**Figure 1:**
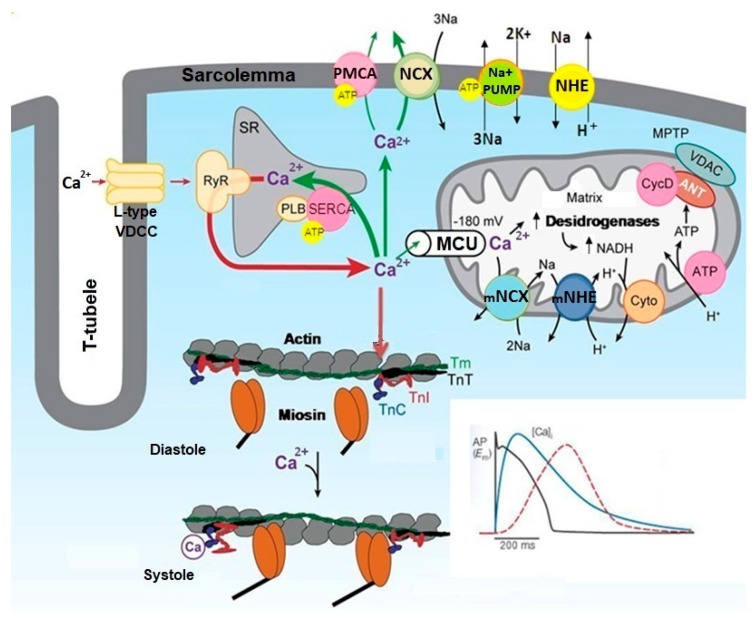
Molecular mechanisms involved in cellular Ca^2+^ homeostasis in cardiac cells. This figure illustrates that Ca^2+^ influx through L-type VDCC stimulates the release of Ca^2+^ from the SR through the RyR, increasing the cytosolic Ca^2+^ concentration ([Ca^2+^]c). Ca^2+^ binds to TnC and promotes the interaction of TnC with TnI, causing TnI to move from the active site of the actin, allowing the displacement of TmT and TnT, and muscle contraction (systole). This increase in [Ca^2+^]c increments the Ca^2+^ influx into mitochondria via MCU, stimulating the synthesis of ATP. The increase of [Ca^2+^]c is restored to basal levels (resting) by Ca^2+^ sequestration SR via SERCA and Ca^2+^ extrusion via PMCA and NCX, and this reduction in [Ca^2+^]c promotes the relaxation of cardiac cells (diastole). The inset represents the electromechanical coupling in the cardiac cell. The initial wave produces a transient increase in cytosolic Ca^2+^ concentration followed by cell contraction. Once the electrical stimulus has finished, the cytosolic Ca^2+^ concentration returns to baseline by the action of mechanisms involved in Ca^2+^ sequestration and extrusion, allowing relaxation of cardiac cells (adapted from Bers, 2008 [5]).

In addition to SR, mitochondria also participate in the cellular homeostasis of Ca^2+^ during transient elevations of [Ca^2+^]c in cardiac cells, contributing to the contraction-relaxation cycle [5]. The mitochondrial membranes are equipped with Ca^2+^ transporter proteins that regulate the Ca^2+^ influx and efflux in mitochondrial matrix, thus adjusting the Ca^2+^ concentration in mitochondrial matrix ([Ca^2+^]m) [11]. In cardiac cells and other excitable cells, Ca^2+^ influx into mitochondria is mainly regulated by mitochondrial Ca^2+^ uniporter (MCU) [12], and its efflux is mainly regulated by mitochondrial Na^+^/Ca^2+^ exchanger (mNCX) [13,14]. Thus, the participation of mitochondria in Ca^2+^ homeostasis in cardiac cells has important implications in the cardiac cycle and contraction-relaxation process. Figure 1 illustrates the molecular mechanisms involved in cellular Ca^2+^ homeostasis in cardiac cells.

### 1.2. Cardiac Dysfunctions Produced by Ischemia and Reperfusion

**Figure 2:**
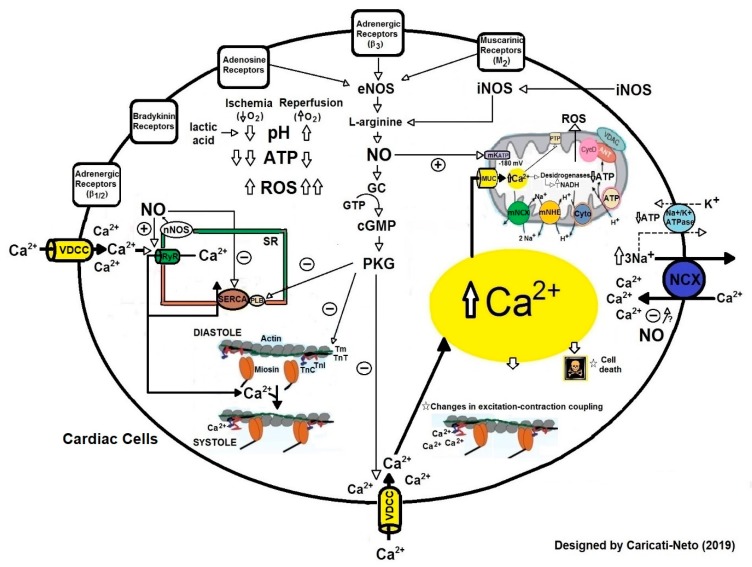
Molecular mechanisms involved in cellular dysfunctions caused by ischemia and reperfusion (I/R) injury in cardiac cells. During ischemia, intracellular accumulation of inorganic phosphate, lactate and H reduce intracellular pH, which increases NHE activity. ATP deficiency reduces the activity of ATP-dependent ion transport proteins, such as Na^+^/K^+^-ATPase, PMCA and SERCA, favoring Na^+^ and Ca^2+^accumulation in the cytosol, which interferes with the plasma membrane NCX activity, worsening the cytosolic Ca^2+^ overload. The Ca^2+^accumulation in cytosol compromises the mitochondrial membrane potential, leading to increased mitochondrial Ca^2+^ uptake through the MCU, generating mitochondrial Ca^2+^ overload. This Ca^2+^ overload collapses mitochondrial function, altering ATP production. The cytosolic and mitochondrial Ca^2+^ overload alters excitation-contraction coupling, and thus increasing the incidence of cardiac arrhythmias. In reperfusion, the cytosolic and mitochondrial Ca^2+^ initiated in ischemia, additionally compromise oxidative phosphorylation and produce increment in the permeability of the internal mitochondrial membrane. This results in the production of free radicals that stimulate the opening of transition pores of mitochondrial permeability, releasing cytochrome C, which activates the apoptotic cascade. Increased production of free radicals causes structural damage and aggravates the cytosolic Ca^2+^ overload, causing severe arrhythmias and cardiac collapse.

The molecular mechanisms involved in regulation of [Ca^2+^]c in cardiac cells are responsible for fine control of the heart rate. Thus, changes in Ca^2+^ extrusion or buffering may precipitate the spontaneous release of Ca^2+^ from the SR, and generate triggered activity as the delayed after depolarization [15]. During ischemia, the deregulation in cellular Ca^2+^ homeostasis caused by inadequate functioning of Ca^2+^-ATPases results in cytosolic and mitochondrial Ca^2+^ overload, collapsing the mitochondrial function and ATP production [16]. During reperfusion, this Ca^2+^ overload is aggravated due to increased Ca^2+^ influx into cytosol through NCX activity and the increment in formation of free radicals [16]. The increment in the formation of free radicals causes oxidation of structural proteins and proteins involved in the respiratory chain, oxidation of pyridine nucleotides, changes in the permeability of internal mitochondrial membrane, decoupling of oxidative phosphorylation, and a collapse in ATP production by mitochondria [17]. Figure 2 illustrates the molecular mechanisms involved in cellular dysfunctions caused by ischemia followed by reperfusion (I/R) injury in cardiac cells.

## 2. Cardioprotective Strategies Against Myocardial Lesions Caused by I/R Injury

Although the reperfusion is the main treatment of acute myocardial infarction, this process may aggravate the myocardial lesions caused during ischemia, generating severe cardiac collapse [18,19]. As this collapse can be fatal, several non-pharmacological and pharmacological cardioprotective strategies have been proposed to protect the myocardium of lesions caused by I/R injury and increase the effectiveness of IHD treatment. In this paper, we analyze the recent advances in non-pharmacological and pharmacological strategies of cardioprotection.

### 2.1. Non-Pharmacological Strategies for Cardioprotection

A few decades ago, it was discovered that cardiac cells possess intracellular signaling pathways that, when stimulated, protect these cells against the damage caused by I/R injury. This ability of cardiac cells to protect themselves against injury has been termed “cardioprotection” [20]. Cardioprotection can be stimulated by several non-pharmacological procedures, including ischemic pre- and postconditioning applied directly in myocardium [20]. The conditioning phenomena exert powerful cardioprotection but still use I/R, which as such is injurious, and in pre- and postconditioning, there is also the risk of atherosclerotic lesions and coronary microembolization, particularly in ischemic postconditioning [20]. Thus, a better understanding of the signal transduction involved in conditioning phenomena may help to stimulate the cardioprotective response without the coronary lesions and inevitable myocardial injury caused by I/R, and additionally, may help to account for and attenuate interference from confounding risk factors, comorbidities, and comedications [20]. Cardioprotective response also can be stimulated by brief cycles of I/R at a remote non-cardiac site (remote ischemic conditioning) [20]. Cardioprotection stimulated by remote ischemic conditioning has been established in many experimental studies and successfully translated to patients [20]. This cardioprotective strategy which is characterized by a systemic response, efficiently reduces the infarct size and improves the prognosis for reperfused myocardial infarction [20].

In addition, cardioprotective response can also be stimulated by exposure to different degrees of hypoxia (as well as by a subhypoxic drop in oxygen tension), and by both hyper- and hypothermia [20]. The cardioprotective effect of hypothermia has been well demonstrated in animal models of acute myocardial infarction [20]. This cardioprotective strategy has been associated with prevention of the no-reflow phenomenon and long-term improvement in terms of left ventricular remodeling and performance [20].

Interestingly, recent evidence indicates that some physiological and pathological conditions, such as the regular practice of physical exercise and sympathetic hyperactivity, can potentially stimulate the cardioprotective response. Although the signal transduction involved in this response is still not known, these conditions can virtually stimulate the same cellular signaling pathways involved in conditioning. In this paper, we analyze the cardioprotection stimulated by ischemic pre- and post-conditioning, remote ischemic conditioning, hypothermia, regular practice of physical exercises and sympathetic hyperactivity.

#### 2.1.1. Cardioprotection Stimulated by Ischemic Preconditioning

Ischemic preconditioning is when cardioprotective response is stimulated by brief episodes of I/R applied prior to sustained metabolic stress (including ischemia and I/R) [1,20]. This procedure reduces the number of premature ventricular beats, and the incidence and duration of ventricular tachycardia and fibrillation [18,19,20,21,22,23,24,25]. Ischemic preconditioning activates cellular survival pathways mediated by phosphatidyl inositol 3′-hydroxy kinase (PI3K)/protein kinase B (Akt) and increases cytosolic levels of nitric oxide (NO) in cardiac cells, stimulating cardioprotection (see Figure 2) [20]. During ischemic preconditioning, mitochondrial ATP-dependent K^+^ channels (mK_ATP_) located on the inner mitochondrial membrane of cardiac cells are activated, increasing mitochondrial matrix volume, fatty acid oxidation, and production of free radicals [26,27]. These channels control the mitochondrial volume through the K^+^ fluxes and oxidation of fatty acids involved in the cellular response stimulated by ischemic preconditioning [26,27]. The opening of mK_ATP_ channels involves the epsilon isoform of protein kinase C (PKCε) [26,27], impeding the hyperpolarization of inner mitochondrial membranes [28]. This avoids excessive mitochondrial Ca^2+^ accumulation, which prevents the prolonged opening of mitochondrial permeability transition pore (MPTP), and induces cytochrome C release and caspases activation [28,29,30,31]. The activation of the mK_ATP_ channels stimulated by ischemic preconditioning is preceded by the activation of other kinases, such as tyrosine kinases and mitogen-activated protein kinases (MAPK), which activate the NO-inducible synthase (iNOS), and NO synthesis. NO activates intracellular signaling mediated by guanylate cyclase (GC)/cyclic guanosine monophosphate (cGMP)/protein kinase G (PKG) pathway and stimulates the opening of mK_ATP_ decreasing mitochondrial Ca^2+^ overload and mitochondrial collapse in cardiac cells, thus inducing cardioprotection [32]. Our studies showed that ischemic preconditioning significantly reduced the incidence of severe arrhythmias and lethality caused by cardiac I/R injury mainly due to the attenuation of excitation-contraction decoupling resultant from the ionic imbalance in the cardiac cells [33].

**Figure 3:**
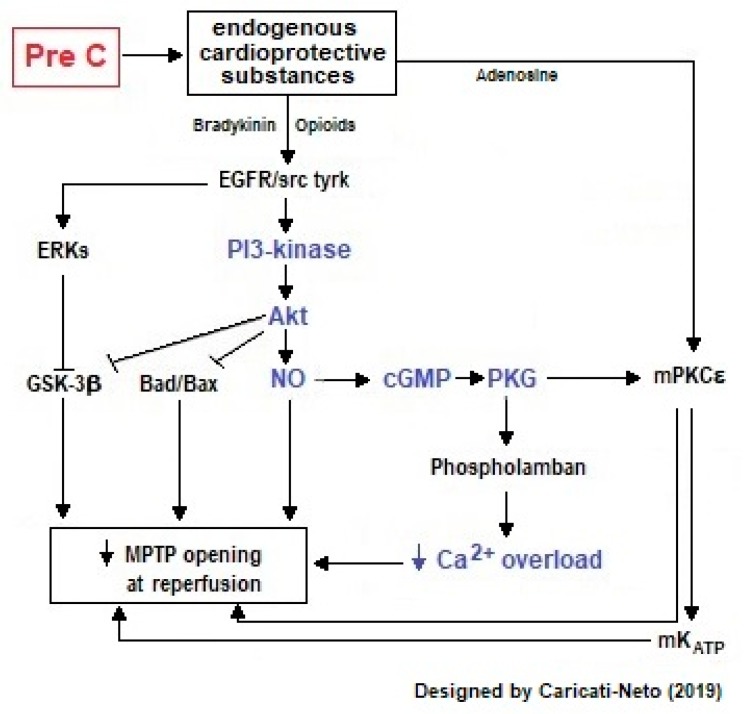
Intracellular signaling involved in cardioprotective response stimulated by ischemic preconditioning (PreC) in cardiac cells. This figure illustrates that PreC activates cellular survival pathways mediated by PI3K/Akt and increases cytosolic levels of NO in cardiac cells. NO activates intracellular signaling pathway mediated by GC/cGMP/PKG and stimulates the opening of mK_ATP_ by decreasing mitochondrial Ca^2+^ overload. These cellular responses attenuate cytosolic Ca^2+^ overload, reduce the formation of free radicals, prolonged opening of MPTP channels, and increase ATP production, attenuating excitation-contraction decoupling, and thus reducing the incidence of severe arrhythmias and cellular death. The arrows represent activation signal and “T” bars represent inhibition signal.

The main advantages of ischemic preconditioning are that this process can be induced by ischemia directly in the heart or remotely, and can be programmed in the case of surgical interventions or angioplasty—situations in which the function of the heart is compromised [23,24,25]. Ischemic preconditioning has two periods of cardioprotective efficacy with different characteristics: (1) the first window of protection (early preconditioning) is more effective in reducing the size of the infarct, and (2) the second (late) protection window is more effective against “myocardial stunning” [23,24,25]. Figure 3 illustrates intracellular signaling involved in cardioprotective response stimulated by ischemic preconditioning in cardiac cells.

#### 2.1.2. Cardioprotection Stimulated by Ischemic Postconditioning

Ischemic postconditioning is when cardioprotective response is stimulated by brief episodes of I/R applied after the main insult [1,20,33,34]. This procedure reduces the size of the infarcted cardiac area to a level equivalent to ischemic preconditioning [35,36]. This cardioprotective response stimulated by ischemic postconditioning is associated with improved endothelial function, reduction in superoxide generation, and a reduction in apoptosis rate and microvascular lesions [34]. Cardioprotection stimulated by ischemic postconditioning is mainly mediated by activation of intracellular signaling pathways involved in the survival of cardiac cells [20]. The mechanisms by which ischemic postconditioning confers cardioprotection against cardiac I/R injury resemble those of ischemic preconditioning; where adenosine has a cardioprotective effect conferred by ischemic postconditioning, and adenosine inhibitors may eradicate this effect. Other mechanisms include the participation of endothelial NO synthase (eNOS), NO, GC, mK_ATP_ channels and MPTP [34]. Thus, drugs that inhibit NO biosynthesis (Nω-nitro-L-arginine methyl ester, L-NAME) or blockade the cellular action of NO (GC inhibitors) reduce the cardioprotective effect triggered by ischemic postconditioning [37,38].

Ischemic postconditioning is more clinically applicable than ischemic preconditioning, since its application does not occur before an ischemic episode, but at the time of reperfusion [35,36]. In human studies, electrocardiographic parameters and ischemic myocardial perfusion were improved through ischemic postconditioning (2 cycles of 90s separated by 3 and 5 min) in patients who suffered acute myocardial infarction and who underwent reperfusion therapy [39]. Some studies showed that patients submitted to coronary angioplasty to treat acute myocardial infarction were submitted to ischemic postconditioning with 4 cycles of I/R (1 min each) [40]. This procedure produced a reduction in serum levels of creatine kinase (CK), improvement of microvascular circulation and reduction in the infarct area size [40]. The main advantages of ischemic postconditioning are its applicability in patients who have suffered unpredictable acute myocardial infarction and the possibility of short-term treatment in the post-ischemic phase [35,36].

**Figure 4:**
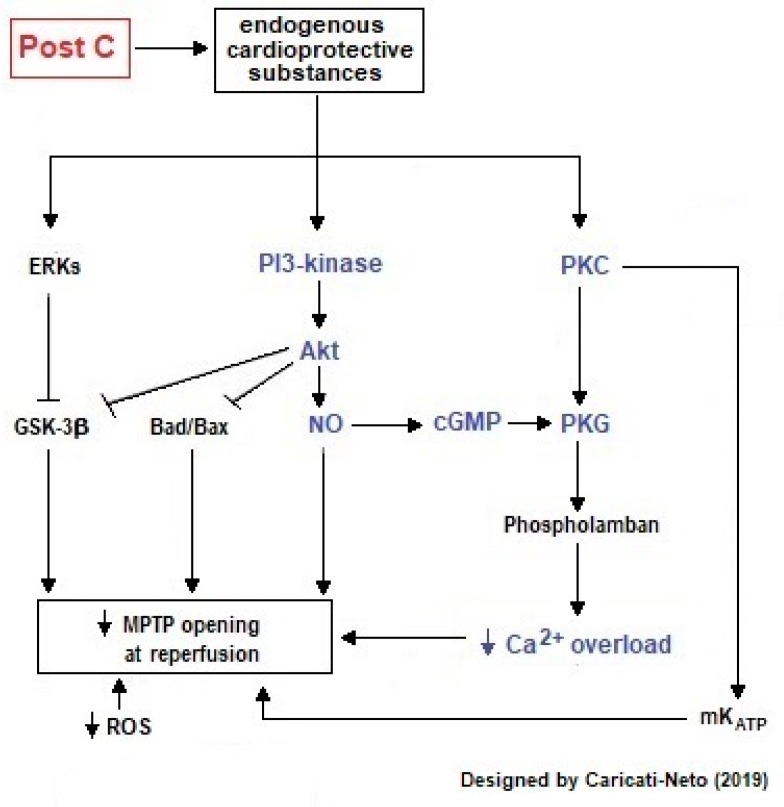
Intracellular signaling involved in cardioprotective response stimulated by ischemic postconditioning (PostC) in cardiac cells. This figure illustrates that PostC activates cellular survival pathways mediated by PI3K/Akt and increases cytosolic levels of NO in cardiac cells. This increase in NO biosynthesis activates intracellular signaling mediated by GC/cGMP/PKG pathway that produces increments in the activity of mK_ATP_ channels, preserving the mitochondrial bioenergetics, and preventing the prolonged opening of the MPTP. The arrows represent activation signal and “T” bars represent inhibition signal.

Studies in animal models of myocardial infarction showed that ischemic postconditioning improved myocardial metabolic recovery and increased myocardial salvage [41,42]. Our studies in animal models of cardiac I/R injury indicate that ischemic postconditioning attenuates ionic imbalance in cardiac cells by reducing the cytosolic and mitochondrial Ca^2+^ overload, thus reducing excitation-contraction decoupling and incidence of severe arrhythmias and lethality caused by I/R injury [42]. Ischemic postconditioning also reduces the formation of free radicals, prolonged opening of MPTP, and ATP deficit production [41,42]. Our studies also indicate that the cardioprotection stimulated by ischemic postconditioning is less efficient than that induced by ischemic preconditioning [42]. In contrast to the beneficial effects observed in animal models, ischemic postconditioning in human using surrogate markers of myocardial salvage has yielded conflicting results [41]. Figure 4 illustrates intracellular signaling involved in the cardioprotective response stimulated by postconditioning in cardiac cells.

#### 2.1.3. Remote Ischemic Conditioning

Several studies have shown that a cardioprotective response can also be stimulated by brief cycles of I/R at a remote non-cardiac site [43,44,45,46,47,48]. This cardioprotective strategy known as remote ischemic conditioning was initially regarded as a laboratory curiosity and somewhat neglected, but gained popularity when it was stimulated from organs remote from the heart, and was later successfully translated to humans [43,44,45,46,47,48]. Remote conditioning by short repetitive cycles of I/R on an extremity reduces infarct size and improves the prognosis of patients with reperfused myocardial infarction [44,46]. In addition, it also protects the vasculature and various other parenchymal organs from I/R injury [44,46]. With respect to the time frame, remote ischemic conditioning can be classified as pre- (before the target organ ischemia), per- (during the target organ ischemia) or post- (after the target organ ischemia) conditioning [45,46,47,48]. When the time lag between the remote conditioning stimulus and the target ischemia is of longer duration than minutes, there is delayed remote ischemic conditioning [45,46,47,48]. The intramyocardial signal transduction of remote conditioning appears to be very similar to that of local conditioning, however, the transfer signal from the remote tissue or organ to the heart or other target organs is still enigmatic [47,48].

Remote ischemic conditioning is obviously a systemic response and the details of the signal transduction involved in this response are largely unclear. However, it has been proposed that signal transduction in remote ischemic conditioning in the myocardium is remarkably similar to that of local ischemic pre-and postconditioning [47,48]. Neuronal and humoral mechanisms are involved in the signal transfer from the peripheral stimulus site to the heart [47,48]. They act in concert and interact on three different levels: stimulus, systemic and target organ. At the stimulus level, peripheral sensory nerves are directly activated and humoral factors are released that subsequently activate the peripheral sensory nerves [47,48]. The importance of these nerves in cardioprotection stimulated by remote ischemic conditioning was evidenced by the loss of this response through the peripheral nerve transection or local anesthesia [48]. At the systemic level, peripheral sensory afferent nerves travel to the spinal cord, project into automonic centers of the central nervous system and this results in stimulation of efferent vagal nerves [47,48]. This vagal stimulation releases acetylcholine to activate muscarinic receptors in the target and non-target organs [47,48]. Activation of muscarinic receptors in non-target organs stimulates the release of humoral factors, which subsequently act on the target organs [47,48]. In the target organ, the stimulation by humoral factors of intrinsic autonomic nerves and intracardiac ganglia, activates different classes of receptors and intracellular signaling pathways involved in cardioprotection [47,48]. In fact, remote ischemic conditioning can be abolished by the blockading of ganglionic nicotinic receptors by hexamethonium [48] The blockading of these receptors inhibits impulse transmission from preganglionic to postganglionic neurons of the sympathetic and parasympathetic autonomic system, reducing the activation of cardiac adrenergic and cholinergic receptors [48]. These receptors are involved in the cardioprotective responses [48].

A variety of remote stimuli elicit target organ protection, including electrical stimuli such as transcutaneous electrical nerve stimulation, pharmacological stimuli such as adenosine and bradykinin, mechanical stimuli such as trauma, and cycles of I/R on an extremity or organ. Studies in humans and laboratory animals showed that cardioprotective humoral factors can be detected in systemic circulation after cycles of transcutaneous electrical nerve stimulation, and that trauma by transverse abdominal skin incision reduced myocardial infarct size [48]. In addition, the release of humoral cardioprotective factors in response to remote ischemic conditioning was significantly reduced by the blockade of adenosine receptors produced by intra-arterial caffeine infusion [48]. These findings indicate that remote ischemic conditioning can stimulate the cardioprotective response.

#### 2.1.4. Hypothermia

Studies using animal models showed that mild therapeutic hypothermia is a powerful cardioprotective strategy, reducing myocardial infarct size, reducing the no-reflow phenomenon, and improving healing after infarction [48,49,50]. In these experimental studies, various cooling techniques were tested including whole-body hypothermia, synchronized hypothermic coronary venous retro-perfusion, heat exchangers, and regional hypothermia targeting the heart alone [48,49,50]. However, in humans, the most widely used techniques are surface cooling and cooling by endovascular heat-exchange catheters [48,49,50]. Clinical studies suggest a lack of benefit of this procedure in patients presenting acute myocardial infarction in most trials [20]. The reduction in temperature necessary to produce cardioprotection is mild (32–34 °C), appears to have no detrimental effects on left ventricular function or regional myocardial blood flow, and may improve microvascular reflow to previously ischemic heart tissue [48,49,50]. Experimental and clinical studies have shown that for therapeutic hypothermia to be effective it must be initiated as early as possible after the onset of ischemia, and initiated before reperfusion [48,49,50]. The mechanisms involved in cardioprotective response stimulated by hypothermia have yet to be conclusively determined but may include a decrease in tissue metabolic rate, preservation of high energy phosphates, a reduction in tissue apoptosis or induction of heat shock proteins.

#### 2.1.5. Physiological and Pathological Conditions that Potentially Stimulate the Cardioprotective Response

Several studies have indicated that regular practice of physical exercise constitutes a physiological condition that can potentially stimulate the cardioprotective response [51,52,53]. It is well established that moderate exercise is an effective and economic way to prevent and treat cardiovascular diseases, because exercise induces cardiac hypertrophy and neo-angiogenesis, essential determinants for cardioprotection [51,52,53]. Similar to ischemic preconditioning, the practice of moderate (1 to 3 episodes of exercises per week) and high-intensity exercise can decrease infarct size following myocardial infarction [51,52,53]. It has been proposed that moderate exercise induces the intracellular IGF-1-PI3K-Akt, NO, C/EBPβ, and PGC-1α signaling pathways, promoting protection against cardiac ischemic disease, cardiac aging and cardiac metabolic derangement [54]. One mechanism involved in this process is the upregulation of eNOS activity and action in vascular endothelium, which contributes to cardioprotection [55]. Patients who practice regular exercise have more red blood cells with functional eNOS, which contributes to systemic nitrite homeostasis and cardioprotection [55]. These findings indicate that the regular practice of physical exercise can potentially stimulate the cardioprotective response.

Some lines of investigation suggest that sympathetic hyperactivity associated to arterial hypertension constitutes a pathological condition that can potentially stimulate the cardioprotective response [42,56,57,58]. Studies performed in humans and animal models of primary arterial hypertension showed that this cardiovascular disease is associated with increased peripheral vascular resistance, progressive cardiac hypertrophy and heart failure, and sympathetic hyperactivity [56]. Spontaneously hypertensive rats (SHR) represent the main animal model of study of human arterial hypertension [56]. In these animals and humans, the arterial hypertension causes a significant reduction in microvascular density and blood supply in several organs, including the heart [56]. The reduction in the density of myocardial capillaries, and dysfunction in the process of angiogenesis during the development of hypertension leads to moderate ischemia [56]. Other important factor that contributes to this reduction of microvascular density is apoptosis of endothelial cells caused by increased oxidative stress [56]. Some studies have proposed that the moderate ischemia associated to hypertension can stimulate cardioprotective response, mimetizing the ischemic preconditioning [33,34,39,40,42,57,58]. Our studies conducted in SHR have shown a reduced incidence of arrhythmias and lethality when this animal model of hypertension was submitted to cardiac I/R injury, similar to that previously described with the animals treated with ischemic pre- and postconditioning during cardiac I/R injury [33,42]. These findings suggest that the sympathetic hyperactivity associated to hypertension can potentially stimulate the cardioprotective response.

### 2.2. Pharmacological Strategies of Cardioprotection

As mentioned above, cardiac cells possess intracellular signaling pathways that, when stimulated, protect these cells against the damage caused by I/R injury. However, this ability of cardiac cells to protect itself against injury can be stimulated by several classes of pharmacological agents, including catecholamines, purines (adenosine), opioids, endothelin, angiotensin, bradykinin, acetylcholine, testosterone, estrogens, adrenomedullin, phenylephrine, and others [20,59,60]. This pharmacological cardioprotection is mainly mediated by activation of cell receptors and intracellular signaling pathways involved in the survival of cardiac cells [20]. On this topic, we analyze the cardioprotective effects stimulated by agonists of β-adrenoceptors (β-AR) or adenosine receptors, L-type VDCC or MCU blockers, modulators of NO biosynthesis or NCX, resveratrol, methylene blue and intestinal lipase inhibitors.

#### 2.2.1. Cardioprotection Stimulated by Agonists of β-AR

In arterial hypertension in humans and in laboratory animals (SHR), an association between the reduction of angiogenesis and the low expression of the VEGF receptor type II (KDR) has been shown [61,62]. In addition, the hyperactivity of cervical, splanchnic and renal sympathetic neurons was shown in these models of hypertension, suggesting that this cardiovascular disease is intimately related to sympathetic hyperactivity [63,64,65,66]. Our studies using SHR showed that this animal model of arterial hypertension presents reduced incidence of arrhythmias and lethality when submitted to cardiac I/R injury [42]. These findings suggest that the cardioprotective response observed in hypertensive animals can be directly associated to sympathetic hyperactivity [41].

Several studies have shown that non selective and selective agonists of β-AR stimulate cardioprotection against cardiac I/R injury [67,68,69]. It was shown that treatment with non selective agonists β-AR (isoproterenol) and selective agonists of β_1_-AR (denopamine), β_2_-AR (formoterol and clenbuterol) and β_3_-AR (BRL37344) reduced ventricular arrhythmias and infarct size in laboratory animals submitted to cardiac I/R injury [67,68,69]. A single dose of BRL37344 before reperfusion improved long-term left ventricular function in animals submitted to cardiac I/R injury [69]. Activation of β_3_-AR stimulates cellular survival pathways mediated by PI3K/Akt and increases cytosolic levels of NO in cardiac cells [69]. This increase in NO biosynthesis activates intracellular signaling mediated by the GC/cGMP/PKG pathway, which produces an increment in the activity of mKTP channels, preserving the mitochondrial bioenergetics, attenuating excitation-contraction decoupling, and thus reducing the incidence of severe arrhythmias and cellular death (see Figure 2) [67,68,69]. In addition, activation of β_1/2_-AR stimulates the increment of intracellular cAMP concentration in cardiac cells, which in turn, increases the cAMP efflux mediated by multidrug resistance protein transporters and elevates the extracellular cAMP concentration and adenosine, thus culminating in activation of adenosine receptors [42]. These cellular responses mediated β-AR reduce cytosolic Ca^2+^ overload, oxygen consumption and ATP deficit [67,68,69,70].

Our studies have showed that the activation of cardiac β-AR with isoproterenol stimulates the cardioprotective responses in rats submitted to I/R injury, similar to that observed in hypertensive animals [42]. In addition, our studies have showed that these cardioprotective response stimulated by agonists of β-AR was totally eliminated by pretreatment of animals with antagonist of β_1_-AR (atenolol) [42]. These findings support the direct involvement of β-AR in the cardioprotective response. Thus, this response can be pharmacologically stimulated by agonists of these receptors.

#### 2.2.2. Cardioprotection Stimulated by Agonists of Adenosine Receptors

Adenosine generated by ATP hydrolysis exerts its biological effects through interaction with P_1_ (adenosine receptors), which are divided into four sub-types of metabotropic receptors, named A_1_, A_2a_, A_2b_ and A_3_ [71]. The A_1_ receptors are involved in the regulation of the heart and renal function and vascular tone (vasoconstriction), while the A_2a_ and A_2b_ receptors participate in the cardioprotection process [72,73]. It was shown that adenosine induces NO release, increasing the release of cysteinyl leucotrienes and stimulating the cardioprotective response [74]. The NO production is conditioning with protein S-nitrosylation (SNO), another critical component of cardioprotection mediated by NO in ischemic pre- and postconditioning [75]. These finding support the direct involvement of adenosine receptors in cardioprotective response. Thus, this response can be pharmacologically stimulated by agonists of these receptors.

#### 2.2.3. Cardioprotection Stimulated by Blockers of L-type VDCC

The VDCC blockers constitute a group of drugs clinically used in the treatment of cardiovascular diseases, which include cardiac arrhythmias, Prinzmetal’s angina, and arterial hypertension. These drugs promote vasodilation, reduction of heart rate, reduction of atrioventricular conduction and arrhythmias [76]. There are three classes of L-type VDCC blockers: dihydropyridines, benzothiazepines and phenylalkylamines [76].

Dihydropyridines (nifedipine, amilodipine, felodipine, isradipine, lercanidine, manidipine, nicardipine, nitrendipine, nimodipine and nisoldipine) and benzothiazepines (diltiazem) preferentially act on the VDCC in smooth muscle cells, promoting vasodilation and reduction in peripheral vascular resistance [76]. Phenylalkylamines (verapamil) preferentially act on the VDCC in cardiac tissue and the conduction system, reducing cardiac activity [77]. Nifedipine is clinically used as antihypertensive, vasodilator and tocolytic agent, and is indicated for the treatment of systemic and pulmonary arterial hypertension, and stable angina pectoris [76,77]. In animal models of cardiac I/R injury, administration of nifedipine before ischemia (10–15 min) reduces the incidence of ventricular tachyarrhythmias, ventricular fibrillation, and lethality [78,79,80,81]. Nicardipine and nimodipine also reduce the incidence of arrhythmias in different models of cardiac I/R injury when given prior to ischemia [80].

Our studies have showed that treatment with nifedipine before ischemia or reperfusion reduces the incidence of ventricular arrhythmias, atrioventricular blockade and lethality in animals submitted to cardiac I/R injury due to attenuation in cytosolic Ca^2+^ overload in cardiac cells [81,82]. When administrated before reperfusion, this cardioprotective effect of nifedipine was lower in comparison to administration before ischemia [81,82]. This result is due to cytosolic Ca^2+^ overload generated during ischemia and increased depolarization of cardiomyocytes at the onset of reperfusion, which reduces the cardioprotective efficacy of nifedipine [81,82,83,84,85]. Clinical studies suggest that post-ischemia treatment with nifedipine in patients with acute myocardial infarction reduced the incidence of ventricular arrhythmias, but was unable to reduce lethality [86,87,88,89,90,91]. In vitro studies conducted in isolated rat heart showed that cardiac arrhythmias produced by oxygen deprivation (30 min) followed by reoxygenation (45 min) were prevented by the treatment of the heart with L-type VDCC blockers [90]. These findings suggest that L-type VDCC blockers produce cardioprotective effects.

#### 2.2.4. Cardioprotection Stimulated by Inhibitors of MUC

Mitochondria are decisive for cellular survival and death. These organelles play a crucial role in the maintenance of ionic gradients and excitation-contraction coupling in cardiac cells because they provide energy, as well as acting as key organelles in the regulation of intracellular levels of Ca^2+^ [16]. In mammalian cardiac cells, the mitochondrial network occupies 30% of the cellular volume and accounts for 95% of cellular ATP production. The Ca^2+^ influx into mitochondria activates dehydrogenases of Krebs cycle, thus stimulating the production of ATP [92]. In cardiac cells, the MCU is one of the major pathways of Ca^2+^ influx into mitochondria [92], and the opening of this Ca^2+^ channel depends on the mitochondrial membrane potential [92]. In physiological conditions, the Ca^2+^ influx mediated by MCU produces transient increment in [Ca^2+^]m, increasing the activity of dehydrogenases of Krebs cycle, and consequently stimulating the ATP synthesis [16,92,93].

During ischemia, a lack of supply oxygen as an electron acceptor reduces the flow of electrons along the respiratory chain and induces depolarization of the inner mitochondrial membrane, limiting the formation of ATP in cardiac cells [92,93]. This ATP deficit dramatically compromises the ATP-dependent cellular processes, including transmembrane ion transport, resulting in ionic imbalance and consequently cytosolic Ca^2+^ overload [92,93]. This event collapses the mitochondrial function and ATP production due to Ca^2+^ overload in matrix mitochondrial mediated by the increment of Ca^2+^ influx via MCU [92,93]. During reperfusion, the cytosolic and mitochondrial Ca^2+^ overload is aggravated due to alterations in NCX activity and the increment in the formation of free radicals [92,93]. Mitochondrial Ca^2+^ overload caused by I/R interferes in cardiac excitation-contraction coupling, increases the production of free radicals, stimulates the persistent opening of MPTP, and favors the formation of Ca^2+^ phosphate crystals, compromising the integrity of the internal mitochondrial membrane [92,93,94,95]. Thus, attenuation of mitochondrial Ca^2+^ overload prevents cardiac collapse, improves the recovery of contractile function and confers cardioprotection against injuries caused by I/R [96,97,98,99,100].

It was shown that inhibitors of MCU, such as red ruthenium and Ru360, reduce the Ca^2+^ influx in cardiac cells producing cardioprotective effects, due mainly to attenuation in mitochondrial Ca^2+^ overload and preservation of functional integrity of the mitochondria, especially the ATP production [82,92,99,100]. Our studies showed that the treatment of animals submitted to cardiac I/R injury with inhibitors of MUC, before ischemia or reperfusion, reduced the incidence of arrhythmias and lethality due to attenuation in mitochondrial Ca^2+^ overload in cardiac cells [82]. However, administration of high doses of red ruthenium can produce cardiotoxic effects, due to inhibition of Ca^2+^ release from SR, due to interaction of this MUC inhibitor with RyR, tubulin and Ca^2+^-ATPases [82,101,102,103]. These findings indicate that the inhibitors of MCU produce cardioprotective effects.

#### 2.2.5. Cardioprotection Stimulated by Modulators of Plasma Membrane NCX

Oxygen deprivation caused by cardiac I/R injury leads to a deficit in ATP production in cardiac cells due to inhibition of ATP-dependent ion transporters, such as Na^+^/K^+^ ATPase. This inhibition leads to an increase in [Na^+^]c, which acts as driving force to reverse the transport of Na^+^ and Ca^2+^ by plasma membrane NCX, causing cytosolic Ca^2+^ overload, which favors an increase in the incidence of arrhythmias during ischemia and early reperfusion [104]. Treatment with modulators of plasma membrane NCX, such as KB-R794 and SEA 0400, increase contractile recovery after ischemia, reducing cytosolic accumulation of Ca^2+^ and mitochondrial influx of Na^+^ on reperfusion in hearts submitted to cardiac I/R injury [104,105,106].

Low molecular weight heparins (LMWH), such as trisulfated disaccharide derived from heparin (TDH), have antiarrhythmic and cardioprotective properties [105,107,108,109,110,111,112,113,114]. The oligo-H LMWH, or 2-O-desulfated heparin and 3-O (HDSO) given before ischemia, prevents the onset of ventricular arrhythmias and reduces lethality caused by cardiac I/R injury [106,107]. Our studies have shown that the treatment with LMWH, such as enoxaparin, ardeparin and TDH, prevents the electrically-induced arrhythmias in isolated rat right atrium and heart [113]. Modulation of plasma membrane NCX combined with blocking of Ca^2+^ and Na^+^ currents by LMWH attenuates the Na^+^ and Ca^2+^ overload in cardiac cells, reducing the incidence of arrhythmias and lethality caused by cardiac I/R injury [113]. Other mechanisms can be involved in cardioprotective effects of LMWH, including blockade of L-type VDCC and Na^+^ channels [113,114]. These results demonstrate that the modulators of plasma membrane NCX produce cardioprotective effects.

#### 2.2.6. Cardioprotection Stimulated by Modulators of NO Biosynthesis

In cardiac cells, NO is synthetized by action of NOS isoforms on L-arginine. NO participate in cellular Ca^2+^ homeostasis and excitation-contraction coupling, producing cardioprotection [115,116,117,118]. Administration of L-arginine and NO donors (sodium nitroprusside) and arginase inhibitors (N-hydroxy-nor-L-arginine or NOHA) reduces the incidence of cardiac arrhythmias and myocardial lesions caused by I/R injury [119,120]. Administration of L-arginine increases plasma concentration of superoxide dismutase, total thiois and ascorbate, and reduces the plasma concentration of xanthine oxidase and malondialdehyde [117,118]. This cause an increase in the activity of antioxidant enzymes in cardiac cells, attenuating free radical formation stimulated by I/R injury [121].

NO formed from L-arginine regulates different cellular processes by activation of soluble GC, increase in cGMP formation and PKG activation [122]. The PKG attenuates Na^+^ and Ca^2+^ cytosolic overload during reperfusion by phosphorylating of fosfoleman (Na^+^/K^+^ ATPase modulating protein), increasing the Na^+^ efflux via Na^+^ pump, and reduce the Ca^2+^ influx via plasma membrane NCX [122]. This PKG action contributes to the reduction of myocardial contractile dysfunctions and the appearance of arrhythmias caused by cardiac I/R injury [123]. PKG stimulates the opening of mKATP via PKC and inhibits the opening of MTPM, reducing the release of C cytochrome and activation of pro-apoptotic cascades [124]. In the activated state, PKG acts on L-type VDCC, reducing the influx of Ca^2+^ to cytosol and phosphorylates fosfolambam, increasing the sequestration of cytosolic Ca^2+^ by SR via SERCA [125]. PKG phosphorylates TnI reduce the sensitivity of TnT to Ca^2+^ [125] and phosphorylates the titin, reducing contractile tone of cardiac cells [126].

Our studies have shown that the administration of micromolar concentrations of L-arginine or sodium nitroprusside reduces the heart rate and contraction force of the isolated heart ventricle in rats submitted to prolonged tissue hypoxia [81,82,127]. Our studies also showed that administration of L-arginine produces protective actions against lesions by I/R injury in different organs, such as the liver and intestine [128,129]. These findings demonstrate that the L-arginine produces cardioprotective effects.

**Figure 5:**
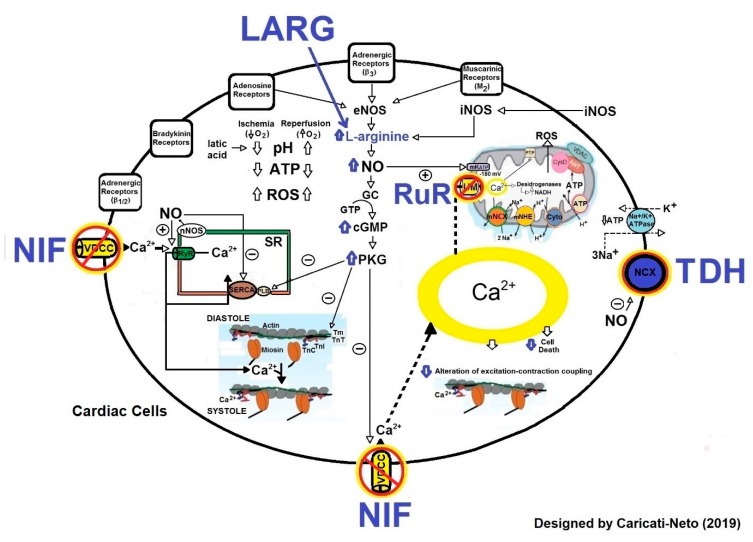
Molecular mechanisms involved in pharmacological cardioprotection. This figure illustrates that the blockade of L-type VDCC by nifedipine (NIF), NCX by TDH, MUC by Ruthenium Red (RuR) and the increase of NO biosynthesis by L-arginine (LARG), produce cardioprotective effects. These effects are resultant of attenuation in cytosolic and mitochondrial Ca^2+^ overload, reduction in mitochondrial collapse, preservation in ATP production, reduction in ROS production and attenuation in excitation-contraction decoupling. The effects of these drugs attenuate or prevent cardiac arrhythmias and cell death caused by cardiac I/R injury. The continuous and dashed arrows represent activation signal and Ө symbol represents inhibition signal.

Figure 5 illustrates the intracellular signaling involved in cardioprotective response stimulated by drugs that modulate the activity of proteins involved in cellular Ca^2+^ homeostasis (L-type VDCC, NCX and MCU) and NO biosynthesis in cardiac cells.

#### 2.2.7. Cardioprotection Stimulated by Resveratrol

Resveratrol (3,5,4’-trihydroxy-trans-stilbene) is a naturally occurring polyphenol antioxidant compound found in Polygonum cuspidatum, grapes, peanuts and berries, as well as their manufactured products, especially red wine, with cardioprotective effects. Resveratrol is a pharmacologically active compound that interacts with multiple targets in a variety of cardiovascular disease models to exert protective effects or induce a reduction in cardiovascular risks [130]. Studies in humans showed that when consumed daily in low concentrations, resveratrol prevents cardiac infarct, improves left ventricular diastolic and endothelial function, reduces the LDL-cholesterol serum levels, and protects against coronary artery disease [131,132].

The cardioprotective effects of resveratrol result from multiple biological actions. This polyphenol exerts protective effects against mitochondrial oxidative stress and apoptosis in neonatal rat cardiac cells induced by hypoxia/reoxygenation injury [133], and increase high-energy compound contents and expression of protein involved in NO pathway in these cells [134,135]. In addition, it decreases catalase synthesis and increases peroxidase and superoxide dismutase activity [136]. Resveratrol reduces the palmitic acid-induced endothelial ROS levels in human aortic endothelial cells. Resveratrol induces endothelial cell autophagy, via AMP-activated protein kinase (AMPK)/mTOR that mediate the effect of resveratrol on ROS reduction [137]. Resveratrol also prevents MPTP opening in myocardial ischemia/reperfusion injury, which was achieved by regulating voltage-dependent anion channel 1 (VDAC1) [138]. The anoxia/reoxygenation injury enhanced VDAC1 phosphorylation, whereas pretreatment with resveratrol dephosphorylated VDAC1 through the Akt-GSK3β pathway, protecting cardiac cells against I/R injury [138]. Other mechanisms are involved in the cardioprotective effects of resveratrol, including the modulation of NHE and attenuation of cytosolic and mitochondrial Ca^2+^ overload [139]. Resveratrol upregulates adiponectin (APN) multimerization in adipocytes, attenuating I/R injury through APN/AMPK signaling [140]. These findings suggest that resveratrol produces cardioprotective effects.

#### 2.2.8. Cardioprotection Stimulated by Methylene Blue

Methylene blue is a redox drug with reported protective effects on mitochondria. The methylene blue ameliorates cyanide toxicity by normalizing the oxidation-reduction state and Ca^2+^ channels function [141]. Methylene blue does not interfere with NOS, but potently inhibits GC, reducing cGMP synthesis and attenuating vascular smooth muscle relaxation [142,143]. The cGMP is an intracellular cardioprotective agent, and its actions account for the low susceptibility to ventricular fibrillation encountered in hearts reperfused after sustained ischemia [143].

Vasoplegia is a term that describes excessive loss of vascular muscle tone leading to a distributive shock status [144]. Vasoplegic syndrome after cardiac surgery is a condition characterized by a hypotension increased cardiac index, low systemic vascular resistance, normal filling pressures, and increased vasopressor and fluid requirements [144]. The use of methylene blue in vasoplegia postcardiac surgery is associated with rapid recovery of hemodynamics, a briefer need for vasopressors, less level mortality, less incidence of renal failure, and shorter length of stay [144].

The methylene blue counteracts the effects of hydrogen sulfide cardiotoxicity by improving cardiac cells contractility and intracellular Ca^2+^ homeostasis disrupted by hydrogen sulfide poisoning [141]. In vivo, methylene blue restores cardiac contractility depressed by sulfide and protects against arrhythmias [141]. The protective effects are mainly due to attenuation in excitation-contraction coupling defects (cellular Ca^2+^ homeostasis and L-type VDCC), reduction in the risk of arrhythmias by stabilization of membrane potential, and preservation of cellular bioenergetics [141]. These finding suggest that methylene blue produces cardioprotective effects.

#### 2.2.9. Cardioprotection Stimulated by Inhibitors of Intestinal Lipase

Metabolic syndrome is a complex disorder represented by a set of cardiovascular risk factors usually related to central fat deposition and insulin resistance, and it is highly associated with cardiovascular disease, increasing overall mortality by 1.5 times and cardiovascular mortality by 2.5 times [145,146,147,148]. In this syndrome, the patient presents comorbidities, for example, dyslipidemia, a disease characterized by the presence of elevated levels of triglycerides (increase in VLDL and LDL-cholesterol) and decreased levels of HDL-cholesterol. Patients with this disease demonstrate alteration in the density and particle size of the lipoprotein, predominantly the type B pattern (small and dense LDL). This association is responsible for the atherogenic character and the inflammatory nature of dyslipidemia and contributes to the increased risk of cardiovascular diseases when compared to those without dyslipidemia and metabolic syndrome [149,150]. The inhibitors of intestinal lipase, such as orlistat, promote body weight reduction, and the consequent weight loss is strongly associated with improvement in blood pressure [149,150]. Our studies have showed that treatment with the inhibitor of intestinal lipase orlistat for 10 days reduced the levels of CK in normotensives rats submitted to cardiac I/R injury [151]. These results may be associated to the effect of orlistat on endothelial function and LDL reduction [151]. However, the treatment with orlistat was not able to reverse the increase in serum CK-MB induced by cardiac I/R injury [151]. Our studies also demonstrated that the treatment for 10 days with orlistat decreased ventricular arrhythmias, atrioventricular blocks and lethality in normotensive rats submitted to cardiac I/R injury, indicating that treatment with orlistat could attenuate or prevent myocardial lesions produced by acute myocardial infarction in humans [151]. These finding indicate that the inhibitors of intestinal lipase produce cardioprotective effects.

## 3. Conclusions

Since the discovery that cardiac cells possess intracellular signaling pathways that, when stimulated, protect these cells against the damage caused by I/R injury, several cardioprotective strategies have been proposed for the treatment of IHD, including acute myocardial infarction. This review focused on the recent advances in non-pharmacological and pharmacological strategies to protect the myocardium of lesions caused by I/R injury. Several non-pharmacological procedures stimulate the cellular signaling pathways involved in the cardioprotective response. In this review, we focused on the cardioprotection stimulated by ischemic pre- and post-conditioning, remote ischemic conditioning, hypothermia, and some physiological and pathological conditions that potentially stimulate the cardioprotective response such as the regular practice of physical exercise and sympathetic hyperactivity. In addition, different classes of drugs stimulate the cellular signaling pathways involved in cardioprotective response. In this review, we focused on the cardioprotection stimulated by agonists of β-AR or adenosine receptors, L-type VDCC or MCU blockers, modulators of NO biosynthesis or NCX, resveratrol, methylene blue and intestinal lipase inhibitors. Although the translation of ischemic postconditioning and remote ischemic conditioning protocols to patients with acute myocardial infarction has been fairly successful, the pharmacological recruitment of cardioprotective signaling has been largely disappointing to date due to the complexity involved in this process. It is important to mention that the most of the cardioprotective drugs are effective when administrated before ischemia, and only in experimental assays performed under controlled conditions. Thus, no definitive human cardioprotective strategy exist.

Although a lot of scientific information about the cellular and molecular mechanisms involved in the cardioprotective responses has been acquired, there are two major outstanding issues to be addressed in the future: (1) understanding the spatiotemporal relationship between chemical messengers and receptors involved in intracellular signaling responsible to cardioprotective response, and (2) devising therapeutic strategies against myocardial diseases based on cardioprotective signaling. Future research is necessary for an adequate understanding of the molecular mechanisms involved in cardioprotection and clinical studies are required to properly test the clinical efficacy and safety of potential cardioprotective strategies.

## Abbreviations

Akt	Protein kinase B
AMPK	AMP-activated protein kinase
APN	Adiponectin
AP	Action potential
AV	Atrioventricular
ATP	Adenosine triphosphate
β-AR	β-adrenoceptors
Ca^2+^	Calcium ion
[Ca^2+^]c	Cytosolic Ca^2+^ concentration
[Ca^2+^]m	Mitochondrial Ca^2+^ concentration
CICR	Ca^2+^-induced Ca^2+^-release
CK	Creatine kinase
ECG	Electrocardiogram
GSK3β	Glycogen synthase kinase 3 β
GC	Guanylate cyclase
cGMP	Cyclic guanosine monophosphate
HDL	High density lipoproteins
HDSO	2-O-desulfated heparin
IHD	Ischemic heart diseases
I/R	Ischemia and reperfusion
LDL	Low density lipoprotein
LMWH	Low molecular weight heparins
L-NAME	Nω-nitro-L-arginine methyl ester
MAPK	Mitogen activated protein kinases
MCU	Mitochondrial Ca^2+^ uniporter
mK_ATP_	Mitochondrial ATP-dependent K^+^ channels
mNHE	Mitochondrial Na^+^/ H^+^-exchanger
MPTP	Mitochondrial permeability transition pore
mTOR	Mammalian target of rapamycin
[Na^+^]c	Cytosolic Na^+^ concentration
NADH	Reduced nicotinamide adenine dinucleotide
NCX	Na^+^/Ca^2+^-exchanger
NO	Nitric oxide
NOHA	N-hydroxy-nor-L-arginine
iNOS	Inducible nitric oxide synthase
eNOS	Endothelial nitric oxide synthase
PI3K	Phosphatidyl inositol 3′-hydroxy kinase
PKA	Protein kinase A
PKC	Protein kinase C
PKG	Protein kinase G
PMCA	Plasma membrane Ca^2+^-ATPase
ROS	Reactive oxygen species
RyR	Ryanodine receptors
SA	Sinoatrial
SERCA	Sarcoendoplasmic reticulum
SHR	Spontaneously hypertensive rats
SNO	S-nitrosylation
SR	Sarcoplasmic reticulum
TnI	Troponin I
TmT	Tropomyosin T
TnT	Troponin T
TDH	Trisulfated dissacharide derived from heparin
VDAC	Voltage-dependent anion channel
VDCC	Voltage-dependent Ca^2+^ channel
VGEF	Vascular endothelial growth factor
VLDL	Very low-density lipoprotein
